# In-person UCLA-PEERS^®^ program for adolescents with autism spectrum disorder in Italy: study protocol for a pilot and feasibility randomized controlled trial with pragmatic features and exploratory EEG markers of treatment response

**DOI:** 10.3389/fpsyt.2026.1988518

**Published:** 2026-10-19

**Authors:** Antonio Narzisi, Maddalena Fabbri-Destro, Elisa Foti, Chiara Cristofani, Gaia Manetti, Ilaria Accorinti, Rosy Muccio, Stefano Berloffa, Pamela Fantozzi, Elena Valente, Valentina Viglione, Pietro Avanzini, Alessandra Carta, Stefano Sotgiu, Emanuele Bartolini, Annarita Milone, Gabriele Masi

**Affiliations:** 1 Department of Child Psychiatry and Psychopharmacology, IRCCS Stella Maris Foundation, Tirrenia, Italy; 2 Consiglio Nazionale Delle Ricerche, Istituto di Neuroscienze, Parma, Italy; 3 Division of Child Neuropsychiatry, University of Sassari, Sassari, Italy

**Keywords:** adolescents, autism spectrum disorder, electroencephalography, in-person intervention, level 1 ASD, N400, Peers^®^, randomized controlled trial

## Abstract

**Background:**

Adolescents with Autism Spectrum Disorder (ASD) Level 1 frequently experience persistent difficulties in peer relationships and social functioning that become more evident as social demands increase. Group-based social skills trainings represent a key evidence-based intervention for this population, yet its availability in routine clinical services is still limited. The Program for the Education and Enrichment of Relational Skills (PEERS^®^) is one of the most empirically supported interventions for autistic adolescents. In Italy, telemedicine delivery has shown promising results, but the effectiveness and implementation of the in-person format in real-world services have not yet been evaluated. In addition, predictors of individual response to psychosocial interventions remain largely unknown.

**Methods:**

This study is designed as a two-arm pilot and feasibility randomized controlled trial incorporating pragmatic features, including delivery within a routine tertiary clinical service, comparison with Treatment as Usual (TAU), and allowance for ongoing concomitant clinical, educational, and pharmacological treatments. It will compare the in-person Italian adaptation of UCLA PEERS^®^ with Treatment as Usual (TAU) at IRCCS Fondazione Stella Maris (Pisa, Italy). Thirty-six adolescents aged 11–13 years with ASD (DSM-5-TR) and Full-Scale IQ >85 will be assessed at baseline (T0) and after the 16-week intervention (T1). Randomization will use block allocation with allocation concealment until completion of baseline assessments; outcome assessors and independent ADOS–BOSCC video coders will remain blind to group assignment. The intervention includes sixteen weekly 90-minute group sessions delivered by certified providers, with parallel caregiver sessions and structured teacher contacts to promote generalization. The primary focus of this pilot trial is feasibility, assessed through recruitment, retention, session attendance, and treatment fidelity. Clinical outcomes will be examined exploratorily to provide preliminary estimates of treatment effects. The QSQ-R will serve as the prespecified principal clinical outcome for estimating change in social functioning from baseline to T1. Parent- and adolescent-report versions will be analyzed separately, considering the Social Initiative and Social Reciprocity dimensions as distinct outcomes rather than deriving a composite total score. Key secondary outcomes include social knowledge (TASSK-R), autism-related social responsiveness (SRS-2), social motivation (SSDS), social anxiety (SAS), and observational change in social-communication behaviors (ADOS–BOSCC). Emotional–behavioral functioning (CBCL; YSR) will be explored as additional secondary outcomes. Baseline electroencephalography (EEG) recorded during a gesture–word integration task will be used to derive the N400 congruency effect and to explore whether individual differences in this measure are associated with treatment response. As a pilot and feasibility trial, the study is not powered for definitive efficacy testing and will estimate feasibility, acceptability, fidelity, and preliminary effect sizes to inform future multicenter trials.

**Discussion:**

This study will provide the first evaluation of in-person PEERS^®^ in the Italian clinical context and test the generalizability of previous telemedicine findings in routine care. The integration of clinical and behavioral outcomes may contribute to mechanism-informed, personalized psychosocial interventions for autistic adolescents and guide the design of future confirmatory studies.

**Trial registration:**

(ClinicalTrials.gov - NCT07207213 – October, 3, 2025).

## 1 Introduction

Autism Spectrum Disorder (ASD) Level 1 is characterized by persistent difficulties in social communication and social interaction in the presence of average cognitive and language abilities (1). During adolescence, these difficulties often become more evident, as social functioning increasingly relies on implicit rules, conversational flexibility, reputation management, and the ability to navigate complex peer networks (2). As a consequence, adolescents with Level 1 ASD frequently experience loneliness, peer rejection, reduced friendship quality, increased social anxiety, and a negative self-concept, all of which may significantly affect their developmental trajectory and quality of life (3). These clinical features highlight the need for structured and evidence-based interventions specifically targeting social competence in ecologically valid contexts (4).

However, the availability of evidence-based psychosocial interventions specifically designed for adolescents with Level 1 ASD is still limited (5). Previous randomized controlled trials on social skills trainings have overall shown positive effects on social functioning and related emotional–behavioral domains, but they are characterized by substantial heterogeneity in terms of intervention models, outcome measures, and individual response to treatment (6). This variability, together with the lack of reliable moderators of treatment outcome, indicates the need for more targeted and ecologically grounded interventions and for the identification of individual predictors of treatment response (5, 7–14). Despite the growing evidence on social skills trainings, three major gaps remain: (a) the heterogeneity of intervention models and outcome measures, (b) the limited availability of effectiveness studies conducted in routine clinical services, and (c) the lack of mechanism-informed predictors of individual response. Addressing these gaps is essential to move from a “one-size-fits-all” approach toward precision psychosocial interventions.

Within this framework, group-based Social Skills Trainings (SSTs) represent one of the most promising approaches for adolescents with Level 1 ASD, as they combine explicit teaching of social rules, behavioral rehearsal, performance feedback, and opportunities for practicing interaction with peers in a structured setting (5, 6, 10). Among the available models, the Program for the Education and Enrichment of Relational Skills (PEERS^®^) is one of the most empirically supported interventions (15, 16). Randomized controlled trials conducted in the United States and in several international cultural adaptations have consistently demonstrated significant improvements in social knowledge, frequency and quality of peer interactions, friendship skills, and reductions in social anxiety, with maintenance of gains over time (17–23). These findings have led to the recognition of PEERS^®^ as one of the most robust evidence-based social skills interventions for autistic adolescents (24).

In Italy, an important step toward the cultural adaptation and dissemination of the model has been represented by a randomized controlled trial evaluating the telemedicine delivery of PEERS^®^, which demonstrated the feasibility and clinical efficacy of the program in the Italian context (25, 26). Although previous studies have suggested that telehealth and in-person delivery of PEERS^®^ may lead to comparable clinical outcomes, this evidence primarily supports telehealth as a strategy to increase accessibility rather than replacing the need for face-to-face implementation (23, 27, 28). The present study is conceived as complementary to this work and aims to extend this line of research by investigating the in-person delivery format. In-person implementation introduces a specific clinical and ecological variable characterized by the physical co-presence of adolescents and caregivers in the same setting, real-time group interaction, *in vivo* behavioral rehearsal, and the direct organization of peer social activities. These elements represent core components of the original PEERS^®^ model and are expected to facilitate the generalization of social skills to everyday contexts (29). Moreover, the in-person format reflects the modality most commonly adopted in routine clinical services, allowing the evaluation of feasibility, acceptability, and implementation indicators in real-world settings (5, 6, 10). In addition to evaluating clinical efficacy, the present study adopts a personalized-medicine perspective by investigating neurophysiological indicators of treatment response. Neurophysiological measures may provide implicit indices of social information processing that are not mediated by linguistic or explicit cognitive strategies. Specifically, the electrophysiological paradigm adopted in the present study is grounded in extensive Event-Related-Potential (ERP) research demonstrating that the N400 component reflects the neural processes underlying semantic integration between multimodal communicative cues (e.g., gestures and linguistic stimuli). In the present protocol, baseline electroencephalography (EEG) will be recorded during a gesture–word integration task modeled on these paradigms to derive the N400 congruency effect as a neurophysiological index of treatment response. In this task, communicative gestures will precede target words that are either semantically congruent or incongruent with the gesture meaning, and the difference in N400 amplitude between these conditions, operationalized as the N400 effect, will be used to identify baseline profiles of semantic integration. The same procedure will be repeated at the end of the PEERS^®^ intervention to assess treatment-related changes in the N400 response. Prior ERP studies (30) suggest that larger N400 congruency effects reflect stronger engagement of semantic integration mechanisms during multimodal processing, and individual variability in these neurophysiological responses may be associated with differential capacity to process social–semantic information relevant to real-world social interaction.

By linking implicit social–semantic processing to behavioral treatment response, the present study adopts a mechanism-informed framework in which individual differences in neurophysiological variability are hypothesized to predict differential clinical outcomes.

Taken together, this study aims to evaluate the effectiveness of an in-person evidence-based social skills intervention for adolescents with Level 1 ASD within the Italian clinical context and to contribute to the identification of objective predictors of treatment response, thereby advancing the development of more individualized and mechanism-informed intervention models. In addition, the trial adopts a mechanism-informed personalized medicine framework aimed at identifying neurophysiological indexes of individual response to a manualized psychosocial intervention.

## 2 Objectives and hypotheses

The primary objective of the present pilot and feasibility randomized controlled trial is to evaluate the feasibility, acceptability, and implementation of the in-person Italian adaptation of the UCLA PEERS^®^ program for autistic adolescents aged 11–13 years within routine clinical services. Feasibility will be assessed through recruitment, retention, session attendance, and treatment fidelity indicators according to the prespecified criteria described below.

A secondary exploratory objective is to estimate preliminary treatment effects of the PEERS^®^ intervention compared with Treatment as Usual (TAU) on social functioning and related clinical outcomes. These analyses are not intended to provide definitive evidence of efficacy but rather to estimate the direction, magnitude, and variability of treatment effects and to inform the design and sample-size requirements of a future adequately powered confirmatory trial. Clinical change will be assessed through a multi-informant battery of standardized measures, complemented by an observational index of social-communication change.

The QSQ-R (16, 31) will serve as the prespecified principal clinical measure for estimating preliminary change in social functioning from T0 to T1. Parent- and adolescent-report versions will be analyzed separately, with Social Initiative and Social Reciprocity treated as distinct outcomes and no composite QSQ-R total score calculated. The ADOS–BOSCC (32) change score will be treated as a key secondary observational outcome, serving as an ecologically grounded clinical anchor for the interpretation of preliminary treatment effects. All other clinical outcome measures will be treated as supportive secondary outcomes.

An additional exploratory and hypothesis-generating objective is to investigate whether electrophysiological indices of social–semantic processing are associated with the magnitude of treatment response. In particular, the N400 congruency effect elicited during the gesture–word integration task will be examined as an exploratory and moderator of clinical change. These analyses are intended to provide preliminary estimates of the direction and magnitude of such associations (30).

These analyses are intended to provide preliminary estimates of the direction and magnitude of such associations. We hypothesize that adolescents receiving the PEERS^®^ intervention will show greater improvements in social functioning from T0 to T1 than those receiving TAU; however, given the pilot nature and sample size of the study, these analyses will be considered exploratory and hypothesis-generating rather than confirmatory. Similarly, analyses examining the association between baseline N400 congruency effects and clinical improvement will be considered exploratory.

Feasibility will be defined *a priori* as recruiting 36 participants within 12 months (average 3 participants/month), retention ≥80% at post-treatment assessment (i.e., ≥29/36 completing T1), and session attendance ≥75%. Treatment fidelity will be considered adequate if ≥80% of core components are delivered according to the adherence checklist.

## 3 Methods

This is a two-arm pilot and feasibility randomized controlled trial with pragmatic features, conducted at IRCCS Fondazione Stella Maris (Calambrone, Pisa, Italy). The pragmatic elements of the study primarily concern its implementation within a routine tertiary clinical service, the use of Treatment as Usual as the comparator, and the allowance and systematic monitoring of concomitant clinical, educational, and pharmacological treatments reflecting usual care. At the same time, the study incorporates explanatory elements, including relatively selective eligibility criteria, delivery by PEERS^®^-certified therapists, structured treatment-fidelity monitoring, and the inclusion of an experimental EEG procedure. Accordingly, the term “pragmatic” is used to describe specific features of trial implementation rather than to characterize the study as a fully pragmatic effectiveness trial.

Participants are assessed at baseline (T0, before treatment) and post-treatment (T1, 16 weeks after T0). Outcome assessors and raters coding the video-recorded interactions are kept blind to group allocation. The trial follows a superiority framework and the pilot and feasibility components will be reported in accordance with the CONSORT 2010 extension for randomized pilot and feasibility trials (33), in addition to the applicable CONSORT recommendations for randomized controlled trials. The protocol is structured in line with the SPIRIT (Standard Protocol Items: Recommendations for Interventional Trials) guidelines (see **Table 1**). A naturalistic follow-up phase is envisioned in future extensions of the project but is not included in the present trial design.

**Table 1:** Schedule of enrolment, interventions, and assessments according to the SPIRIT recommendations.

Study period	Enrolment	Allocation	Baseline (T0)	Intervention period (16 weeks)	Post-treatment (T1)
Enrolment					
Eligibility screen	X				
Informed consent/assent	X				
Baseline characterization					
Diagnostic assessment (ADOS-2)			X		
Cognitive assessment (WISC-IV)			X		
Adaptive functioning (VABS-II)			X		
Demographic and clinical variables			X		
Service-use/concomitant treatments log			X	X	X
Allocation					
Randomization		X			
Interventions					
PEERS^®^ program				X	
Treatment as usual (TAU)				X	
Principal clinical outcome					
QSQ-R			X		X
Key secondary outcomes					
TASSK-R			X		X
SRS-2			X		X
SSDS			X		X
SAS			X		X
ADOS–BOSCC (video-coded interaction)			X		X
Secondary outcomes					
CBCL			X		X
YSR			X		X
Neurophysiological measures					
EEG – gesture–word integration task			X		X

## 4 Study population, recruitment, and eligibility

Participants are adolescents aged 11–13 years (corresponding to the Italian lower secondary school period), a range selected to maximize developmental and school-context homogeneity within treatment groups and to align with the intended target population of the PEERS^®^ for Adolescents program. Eligible participants must have a clinical diagnosis of ASD according to DSM-5-TR criteria (1) and a Full-Scale IQ above 85 on the WISC-IV (34). The FSIQ >85 threshold was selected as an eligibility criterion to ensure a relatively homogeneous cognitive profile and a level of cognitive and verbal functioning considered appropriate for participation in the cognitively and socially demanding activities of the PEERS^®^ program. Importantly, this threshold was not intended as a diagnostic boundary for intellectual disability. Rather, it represents a study-specific eligibility criterion and consequently excludes adolescents with borderline and part of the low-average range of cognitive functioning. All participants must present difficulties in developing and/or maintaining peer relationships and demonstrate sufficient motivation to participate, as assessed during the screen interview, together with at least one caregiver willing to attend parallel sessions. Recruitment occurs through the Day Hospital of IRCCS Fondazione Stella Maris and surrounding territorial services. Exclusion criteria include an FSIQ ≤85, based on the study-specific cognitive eligibility criterion described above, severe psychiatric conditions that would substantially interfere with participation in the intervention (including schizophrenia and bipolar disorder), and clinically significant oppositional or aggressive behavior outside the family context that would interfere with safe group participation. Common psychiatric and neurodevelopmental conditions frequently co-occurring with ASD, including Attention-Deficit/Hyperactivity Disorder (ADHD), anxiety disorders, and Specific Learning Disorders (SLD), are not exclusion criteria and are therefore permitted as comorbid diagnoses. Psychiatric and neurodevelopmental comorbidities are assessed at baseline with clinical assessment by experienced clinicians and review of the available clinical history. Screening and baseline characterization follow PEERS^®^ guidelines and include a face-to-face parent interview to verify feasibility and motivation, followed by a joint entrance interview with parent and adolescent to assess readiness for group intervention (15). Diagnostic phenotyping includes administration of the ADOS-2 module 3 (35) by research-reliable, certified clinicians. Cognitive functioning is assessed with the WISC-IV (34) and adaptive functioning with the Vineland Adaptive Behavior Scales–Second Edition (36). Although the WISC-V is available with Italian standardization, the WISC-IV was retained because, at the time of study implementation, the WISC-V was not yet uniformly available or routinely implemented across all clinical services contributing to participant referral, thereby allowing greater consistency in cognitive eligibility assessment across participants. A standardized video-recorded interaction task is collected at baseline (T0) and repeated at T1 for coding with the Brief Observation of Social Communication Change adapted for adolescents (ADOS–BOSCC; 32), which is used as an observational outcome measure.

### 4.1 Sample size

The target sample size is N = 36 adolescents (18 per arm). As this is a pilot and feasibility randomized controlled trial, the sample size was not determined through a conventional power calculation for detecting treatment efficacy. Instead, it was selected to support the estimation of key feasibility parameters and preliminary variability and effect-size estimates needed to inform the design of a future confirmatory trial, in accordance with methodological recommendations for pilot and feasibility trials (36–38). The planned sample of 18 participants per arm also exceeds the commonly cited rule-of-thumb of at least 12 participants per group for pilot studies when reliable prior information for a formal sample-size calculation is limited (39). Considering the single-center design, recruitment capacity, and the 16-week intervention format, N = 36 was therefore considered both methodologically informative and operationally feasible. The study is not powered for definitive efficacy testing; accordingly, analyses of clinical outcomes will be considered preliminary and hypothesis-generating, with effect estimates and confidence intervals used primarily to inform the design and sample-size calculation of a future multicenter confirmatory trial.

### 4.2 Randomization and blinding

Randomization uses block allocation with a fixed block size of four to maintain balance between arms throughout recruitment. For every four consecutively enrolled participants, two are allocated to PEERS^®^ and two to TAU. The allocation sequence is prepared by an independent researcher not involved in enrolment or outcome assessment. Assignment is disclosed only after completion of baseline assessments to preserve allocation concealment at T0, in accordance with recommended procedures for randomized controlled trials (40, 41). Due to the nature of the intervention, participants and therapists cannot be blinded. However, outcome assessors remain blind to allocation, and families are instructed not to reveal their group during assessments. In particular, the video-recorded interactions used for the ADOS–BOSCC will be coded by independent raters who are blind to group allocation and assessment timepoint. At each assessment point, outcome assessors will be asked to guess group allocation in order to evaluate the integrity of the blinding procedure.

## 5 Interventions

The experimental arm receives the in-person Italian adaptation of the UCLA PEERS^®^ program delivered by certified providers. The intervention is delivered by a team of four therapists, three of whom are formally certified in the UCLA PEERS^®^ for Adolescents program at the Semel Institute for Neuroscience and Human Behavior, University of California, Los Angeles, and one of whom also holds the UCLA PEERS^®^ for Schools certification. To ensure treatment fidelity, a structured session-by-session adherence checklist based on the PEERS^®^ manual is completed by the therapists at the end of each session. The checklist verifies the delivery of the core intervention components, adherence to the standardized session structure, and the review and assignment of homework. Approximately 20% of sessions will be randomly selected, video-recorded and independently rated by a PEERS^®^-trained clinician not involved in treatment delivery to provide an objective measure of treatment fidelity.

Adolescents attend sixteen weekly sessions (approximately 90 minutes each) in closed groups with a maximum of six participants. The 18 participants allocated to the PEERS^®^ arm will be distributed across three closed therapy cohorts of six participants each. Sessions follow a standardized structure combining didactic instruction, Socratic questioning, modelling and role-play, behavioral rehearsal, and feedback (15). Content targets skills required to initiate and maintain peer relationships, including conversational skills, electronic communication, entering and exiting groups, organizing get-togethers, conflict resolution, appropriate use of humor, good sportsmanship, reputation management, and handling teasing and bullying (42).

Caregivers participate in parallel sessions and are trained to act as social coaches, supporting skill acquisition and generalization via structured homework between sessions (e.g., practicing specific social tasks, telephone contact with peers, and planning social activities) (43). Caregivers are expected to attend in person whenever possible; when a caregiver cannot be onsite, participation is maintained via a secure videoconferencing platform to ensure continuity of coaching and adherence to the intervention model. To support ecological generalization to the school context without implementing a full teacher-coaching arm, three structured remote meetings with teachers are scheduled across the 16-week intervention period to share treatment goals, promote consistency between clinical and classroom contexts, and monitor transfer of skills in everyday school interactions (43). Concomitant treatments (e.g., individual therapies, school-based supports, and pharmacological treatments) will be allowed in the experimental arm if they are stable at baseline (i.e., unchanged for at least 4 weeks prior to randomization). Participants receiving psychotropic medications, including stimulants, selective serotonin reuptake inhibitors (SSRIs), and antipsychotics, are eligible for inclusion provided that pharmacological treatment and dosage have remained stable for at least 4 weeks before randomization. Medication use, including drug class and dosage, will be systematically recorded at baseline and monitored throughout the 16-week intervention period. Clinically indicated medication changes during the trial will be permitted and will not result in participant exclusion; any initiation, discontinuation, or dosage modification will be documented. For non-pharmacological concomitant interventions, type and weekly intensity will be systematically recorded throughout the study period. Changes in concomitant pharmacological and non-pharmacological treatments during the trial will be documented and considered in sensitivity analyses where appropriate. The control arm continues Treatment as Usual (TAU), defined as the standard clinical, rehabilitative, pharmacological, and educational supports routinely available through local services. TAU may include individual therapies, school-based support, and psychotropic medication, but it does not include the structured, manualized PEERS^®^ group intervention (44). As in the experimental arm, pharmacological treatment in TAU participants must be stable for at least 4 weeks before randomization. Medication use and any changes in drug or dosage during the 16-week study period will be systematically recorded. Clinically indicated medication changes will be permitted and will not result in exclusion from the study. TAU participants follow the same assessment schedule as the experimental arm. TAU participants follow the same assessment schedule as the experimental arm.

The service-use log will systematically record type, intensity, and duration of all interventions received within TAU, including any social-skills-oriented treatments, in order to verify the absence of structured manualized group social skills trainings comparable to PEERS^®^. TAU intensity will be quantified in terms of weekly hours of intervention and will be considered as a covariate in the statistical models where appropriate.

## 6 Outcomes measures

Outcome measures are collected at T0 and T1. Questionnaires are completed by parents and adolescents and checked for completeness by a researcher who is not involved in treatment delivery. Outcome assessors involved in the ADOS–BOSCC coding remain blind to group allocation and assessment timepoint, and families are reminded at each assessment not to disclose their treatment condition. Outcome measures were selected to capture the proximal targets of PEERS^®^ and clinically meaningful changes in everyday social functioning, combining multi-informant questionnaires with an observational measure of social-communication change. Key secondary and secondary outcomes will be interpreted as supportive and exploratory. To address multiplicity across these outcomes, the Benjamini–Hochberg false discovery rate (FDR) procedure will be applied. The QSQ-R, as the single pre-specified primary outcome, will not be included in this multiplicity adjustment. EEG measures are not considered clinical outcomes but are included both as *a priori* predictors of treatment response within a hypothesis-driven framework and as neurophysiological measures of treatment-related change, assessed by comparing pre-treatment (T0) and post-treatment (T1) measures (see Section 7). **Table 2** summarizes the study outcomes and the specific role of each measure.

**Table 2:** Summary of the primary, key secondary, secondary outcomes and EEG indicators.

Domain	Instrument	Informant	Role in the study
Principal clinical outcome			
Social functioning (peer interactions)	QSQ-R	Adolescent/Parent	Principal clinical outcome^1^
Key secondary outcomes			
Social knowledge	TASSK-R	Adolescent	Key secondary outcome
Social responsiveness	SRS-2	Parent	Key secondary outcome
Social motivation	SSDS	Parent	Key secondary outcome
Social anxiety	SAS	Adolescent	Key secondary outcome
Social-communication change	ADOS–BOSCC	Observation	Key secondary outcome
Secondary outcomes			
Emotional–behavioral functioning	CBCL	Parent	Secondary outcome
Emotional–behavioral functioning	YSR	Adolescent	Secondary outcome
Neurophysiological measures			
Brain-based indicator	EEG (N400)	Neurophysiological	Baseline indicator of treatment response and measure of treatment-related change

^1^ exploratory treatment-effect estimate.

### 6.1 Principal clinical and key secondary outcome measures

The Quality of Socialization Questionnaire–Revised (QSQ-R) (16, 42) is the prespecified principal clinical outcome used to estimate preliminary treatment-related change in social functioning. As this is a pilot and feasibility trial, the QSQ-R analysis is exploratory and is not intended as a confirmatory test of treatment efficacy. It is completed separately by parents and adolescents and assesses social engagement and friendship-related behavior in naturalistic settings, based on the frequency and reciprocity of social get-togethers. It includes two dimensions: Social Initiative, reflecting get-togethers hosted by the adolescent and the number of different peers accepting invitations, and Social Reciprocity, reflecting get-togethers attended as a guest and the number of different peers extending invitations. Higher scores indicate greater frequency and diversity of social engagement. Because the measure consists of count-based indicators rather than a conventional multi-item psychometric scale, internal consistency coefficients are not typically calculated and normative reference scores are not available. For the present study, the Italian-language version previously developed through back-translation and cultural adaptation for the Italian PEERS^®^ study is used. The questionnaire is completed separately by parents and adolescents, and raw scores are used to derive the Social Initiative and Social Reciprocity dimensions for each informant. These dimensions will be treated as separate outcomes, and no composite QSQ-R total score will be calculated. Treatment-related change from T0 to T1 will therefore be examined separately for Social Initiative and Social Reciprocity within the parent- and adolescent-report versions.

### 6.2 Key secondary outcome measures

The Test of Adolescent Social Skills Knowledge–Revised (TASSK-R) (16, 31) is a 30-item criterion-referenced adolescent-report measure assessing knowledge of the social rules and skills explicitly taught during the PEERS^®^ program. Each item presents a sentence stem followed by two response alternatives, from which the adolescent selects the most appropriate response. Correct responses are summed to obtain a total score, with higher scores indicating greater knowledge of PEERS^®^-specific social skills. Because the TASSK-R is a criterion-referenced measure specifically linked to the PEERS^®^ curriculum, it is primarily used to assess acquisition of intervention-specific social knowledge rather than as a norm-referenced measure of general social functioning. No formally validated Italian version of the TASSK-R was available; therefore, an Italian research translation prepared by the research team was used for the present study.

The Social Responsiveness Scale–Second Edition (SRS-2) (45, 46) is a 65-item caregiver-report questionnaire assessing autism-related social difficulties and restricted and repetitive behaviors. Items are rated on a 4-point Likert scale and cover five domains: Social Awareness, Social Cognition, Social Communication, Social Motivation, and Restricted Interests and Repetitive Behavior. Higher scores indicate greater severity of autism-related social difficulties. The SRS has shown strong psychometric properties in Italian samples, including excellent internal consistency (α >.90).

The Stanford Social Dimensions Scale (SSDS) (47, 48) is a 71-item parent/caregiver-report dimensional measure of social functioning. The first 58 items are applicable across language levels, while an additional 13 language-based items are administered to verbal individuals. Items are rated on a 5-point Likert scale and assess five domains: Social Motivation, Social Affiliation, Expressive Social Communication, Social Recognition, and Unusual Approach. The original validation study reported good-to-excellent composite reliability across these domains (range.72–.90). No formally validated Italian version is currently available; therefore, the Italian-language version used in the present study should be considered a translated research version rather than a validated Italian adaptation. No formally validated Italian version of the SSDS was available; therefore, an Italian research translation prepared by the research team was used for the present study.

The Social Anxiety Scale for Adolescents (SAS-A) (49) is an adolescent self-report measure comprising 18 scored items and four filler items. Items are rated on a 5-point scale, with higher scores indicating greater social anxiety. The measure comprises three dimensions: Fear of Negative Evaluation (FNE), Social Avoidance and Distress in New Situations or with Unfamiliar Peers (SAD-New), and Generalized Social Avoidance and Distress (SAD-General). Previous studies have supported its three-factor structure, internal consistency, and construct validity in adolescent samples.

The ADOS–BOSCC Fluent 2 (F2) (32, 50) is an observational measure designed to quantify change in social-communication behaviors in verbally fluent autistic individuals. Standardized video-recorded interactions are coded by trained raters using the BOSCC-F2 coding system, with higher scores reflecting greater severity of autism-related social-communication difficulties. In the present study, video recordings are coded by trained raters blind to group allocation and assessment timepoint. No Italian psychometric adaptation of the BOSCC-F2 is currently available. Use of the BOSCC-F2 in the present trial was authorized by Western Psychological Services (WPS), which provided access to A. Narzisi/IRCCS Fondazione Stella Maris exclusively for use within the present study. A.N. and R.M. completed training for BOSCC-F2 administration and scoring.

### 6.3 Secondary outcome measures

The Child Behavior Checklist for Ages 6–18 (CBCL/6–18) and the Youth Self-Report for Ages 11–18 (YSR/11–18) (51) assess emotional and behavioral problems from the parent and adolescent perspectives, respectively. Both instruments provide syndrome-scale scores and broadband Internalizing, Externalizing, and Total Problems scores, with higher scores reflecting greater emotional or behavioral difficulties. Raw scores are converted to standardized T-scores using appropriate normative references. Italian normative studies have supported the reliability of the ASEBA instruments, with high internal consistency reported for the broadband scales (52).

### 6.4 EEG predictor of outcome (not an outcome)

EEG is recorded during a gesture–word integration task to obtain event-related potentials associated with social–semantic processing. The N400 congruency effect will be used as a neurophysiological index of multimodal semantic processing and examined as a baseline predictor and exploratory moderator of treatment response (see Section 7 and **Table 1**).

## 7 EEG acquisition and role in the study

### 7.1 Stimuli and procedure

Participants will observe short video clips depicting communicative gestures performed by an actor. Gestures will be selected to convey a clear, conventionalized communicative meaning (i.e., emblematic/communicative gestures) and will be presented in randomized order across trials. Each gesture will be followed by the presentation of a written word that can be either congruent or incongruent with the observed action. The critical written word will constitute the time-locking event for ERP analyses. Each trial will begin with a fixation cross, followed by the video stimulus, the word presentation, and an inter-trial interval. Participants will provide an online semantic congruency judgment (congruent vs incongruent) via button press, as in gesture–word semantic integration paradigms (30). Accuracy and reaction times will be recorded for task-engagement monitoring.

### 7.2 EEG recording

Continuous EEG will be recorded using a standard clinical EEG system (Micromed^®^, Mogliano Veneto, Italy), with electrodes positioned according to the International 10–20 System (53).

Data will be recorded with an average reference and re-referenced offline when appropriate.

### 7.3 EEG data analysis

EEG data will be processed offline and segmented into epochs time-locked to the onset of the linguistic stimulus, which constitutes the critical event for the ERP analysis. Epochs will extend from −100 to 1000 ms relative to word onset, and the −100 to 0 ms interval will be used for baseline correction.

Preprocessing will include procedures for the identification and correction of physiological and non-physiological artefacts. Independent Component Analysis (ICA) will be applied to identify components attributable to ocular activity and other stereotyped physiological sources, including cardiac and muscular activity. Following ICA correction, residual epochs will be inspected and rejected when persistent non-physiological artefacts are present.

Event-related potentials will be averaged separately for congruent and incongruent trials. To ensure adequate signal quality and a sufficient number of observations contributing to each condition, a minimum of 50 artefact-free trials per condition will be required for inclusion in the ERP analyses. Participants with fewer than 50 valid trials in either the congruent or incongruent condition will be excluded from the N400 analysis. Given that the task includes 80 trials per condition, this threshold corresponds to retaining at least 62.5% of the available trials in each condition.

The N400 will be operationalized *a priori* as the mean ERP amplitude between 400 and 600 ms after word onset, calculated over a predefined centro-parietal region of interest comprising Cz, CPz, and Pz. The selection of both the temporal window and scalp region was informed by the experimental paradigm on which the present task is based. In the previous adult study using the same gesture–word semantic structure, the congruency-related modulation emerged approximately 428–484 ms after word onset and showed its largest differences over centro-parietal electrodes. The broader 400–600 ms interval was prespecified in the present protocol to encompass the previously observed effect while also taking into account developmental differences in the N400 response. In children, including children with ASD, the N400 may show a later onset, longer duration, and broader scalp distribution than in adults (54).

For each participant, the primary EEG variable will be the N400 congruency effect, calculated as the mean ERP amplitude in the incongruent condition minus the mean ERP amplitude in the congruent condition within the prespecified 400–600 ms interval and centro-parietal region of interest. More negative values will therefore reflect a larger N400 congruency effect. This difference-wave measure is intended to capture individual variability in multimodal semantic processing elicited by the gesture–word incongruency manipulation (30).

The N400 congruency effect will not be treated as a clinical outcome. Rather, it will be examined as an exploratory neurophysiological predictor and moderator of subsequent clinical change. Given the developmental characteristics of the N400 response and the exploratory nature of the present pilot study, interpretation will focus on inter-individual variability in the prespecified N400 measure rather than on the presence or absence of a canonical congruency effect at the group level.

## 8 Statistical analysis plan

Analyses will follow an intention-to-treat approach. Change from T0 to T1 will be examined using linear mixed-effects models with fixed effects for Group, Time, with the Group × Time interaction providing a preliminary estimate of between-group differences in change over time. Participants allocated to PEERS^®^ will receive the intervention across three closed therapy cohorts of six participants each. Although potential within-cohort non-independence is recognized, cohort will not be included as a random effect in the primary model because the variance component would be estimated from only three therapy-level clusters, resulting in an unstable and potentially unreliable estimate. Furthermore, equivalent clustering is not present in the TAU arm. Potential variation across PEERS^®^ cohorts will be examined descriptively and, where estimable, in exploratory sensitivity analyses within the intervention arm. These analyses will be interpreted cautiously given the very small number of cohorts. Covariates such as age, sex, and baseline severity indices may be included where appropriate. Changes over time in the intensity of concomitant treatments, including pharmacological treatment, and TAU will be explored and, where appropriate, included as time-varying covariates in sensitivity analyses.

For the key secondary and secondary clinical outcomes, multiplicity will be addressed using the Benjamini–Hochberg false discovery rate (FDR) procedure. Both unadjusted and FDR-adjusted p-values will be reported, together with effect estimates and 95% confidence intervals. For the QSQ-R, Social Initiative and Social Reciprocity scores will be analyzed separately for parent- and adolescent-report versions. No composite QSQ-R total score will be calculated. Given the exploratory nature of this pilot and feasibility trial, effect estimates and 95% confidence intervals for each QSQ-R dimension and informant will be reported and interpreted as preliminary and hypothesis-generating.

Given the pilot nature of the study and the limited sample size, the Group × Time × N400 congruency effect interaction will be treated as exploratory and hypothesis-generating. The analysis is intended to estimate the direction and magnitude of the association between N400 variability and treatment-related change rather than to provide confirmatory evidence of predictive validity. Effect estimates and 95% confidence intervals will therefore be emphasized, and any observed moderation effect will require replication in an adequately powered independent sample. All statistical analyses will be performed using IBM SPSS Statistics (55).

## 9 Ethics, trial registration, and dissemination

The study has been approved by Institutional Review Board of Meyer Hospital (18.09.2025, protocol number 182/2025). and will be conducted in accordance with the Declaration of Helsinki and applicable regulations. Written informed consent will be obtained from parents or legal guardians and assent/consent from adolescents as appropriate. Patients and the public were not involved in the design of this study. The trial is registered on ClinicalTrials.gov (Identifier: NCT07207213).

As of 4 September 2026, 22 adolescents had been screened, of whom 18 had been enrolled and randomized (9 to the PEERS^®^ intervention and 9 to TAU). The first intervention cycle started as planned on 9 March 2026, and all 18 randomized participants had completed the post-treatment (T1) assessment by the time of manuscript submission. Recruitment remained ongoing, with enrolment continuing toward the prespecified target sample of 36 participants. Completion of recruitment, intervention delivery, and post-treatment assessments is expected by April 2027. Protocol version 1.0, February 2026.

Given the low-risk nature of the behavioral intervention, a formal Data Monitoring Committee was not deemed necessary. This decision is consistent with the low-risk nature of manualized interventions delivered within routine clinical services. Trial conduct, data quality, and protocol adherence will be monitored by the principal investigator and the research team.

No serious adverse events are expected. Any potential adverse events (e.g., psychological discomfort during group sessions) will be recorded and managed according to the standard clinical procedures of IRCCS Fondazione Stella Maris.

All study data will be coded using a unique identification number. Personal information will be stored separately from research data in password-protected files accessible only to authorized members of the research team.

Data will be stored on encrypted institutional servers of IRCCS Fondazione Stella Maris for a minimum of 10 years in accordance with European GDPR regulations and institutional policies.

The final dataset will be accessible only to the principal investigator and authorized researchers involved in the study. This study is funded within the PNRR project “Heterogeneous patterns of clinical onset of the autism spectrum disorder (ASD). A prospective study on putative serum immunological and metabolomic biomarkers of ASD with regressive-onset, pathogenic pathways and clinical-therapeutical implications (PNRR MCNT2-2023-12377178) – funded by the European Union – NextGenerationEU. Results will be disseminated through peer-reviewed publications and scientific conferences, with the aim of supporting evidence-based implementation of PEERS^®^ in Italy and informing future multicenter research, including the potential utility of brain-based features of outcome.

By being conducted within a public tertiary clinical service of the Italian National Health System, this trial will provide information on the real-world feasibility, acceptability, and effectiveness of the in-person PEERS^®^ program in routine care. An anonymized dataset and the statistical code will be made available upon reasonable request in accordance with institutional and GDPR regulations. The integration of neurophysiological indicators and behavioral outcomes is expected to contribute to the development of precision-medicine approaches for psychosocial interventions in autism.

## 10 Limitations

Several limitations of the present study should be acknowledged. First, this is a single-center pilot and feasibility trial with a relatively small sample size. Accordingly, the study is not powered for definitive efficacy testing, and estimates of treatment effects should be considered preliminary and hypothesis-generating. The single-center design may also limit the generalizability of the findings to other clinical and service settings. The FSIQ >85 eligibility criterion further restricts the representativeness of the study sample. Although this threshold was selected to ensure a relatively homogeneous cognitive profile and a level of cognitive and verbal functioning appropriate for the demands of the PEERS^®^ intervention, it is not intended to represent a diagnostic boundary for intellectual disability. The criterion excludes not only adolescents with intellectual disability but also individuals with borderline and some low-average cognitive functioning. Consequently, the findings may not generalize to the broader population of autistic adolescents across the full range of cognitive abilities, particularly those with lower cognitive functioning or higher support needs. In addition, formally validated Italian versions were not available for some secondary outcome measures, including the TASSK-R and SSDS, for which research translations prepared by the study team were used. Although these measures are intended primarily for supportive and exploratory analyses, the absence of formal psychometric validation of their Italian translations should be considered when interpreting findings based on these instruments. A further limitation concerns the potential for contamination in the TAU arm. Although concomitant interventions are documented through a service-use log and participants receiving structured, manualized social-skills programs comparable to PEERS^®^ are excluded, exposure to informal, *ad hoc*, or non-manualized social-skills coaching within routine clinical, educational, or community-based settings cannot be completely ruled out. Such exposure could reduce the observed between-group contrast and should therefore be considered when interpreting preliminary treatment-effect estimates.

Finally, the planned analysis of baseline N400 amplitude as a moderator of treatment response is exploratory and is likely to be underpowered given the limited number of participants in the active arm and the potential additional loss of participants from ERP analyses when fewer than 50 artefact-free trials remain in either condition. Any Group × Time × N400 interaction will therefore be interpreted as hypothesis-generating rather than confirmatory, primarily intended to estimate preliminary associations and inform the design and sample-size requirements of future adequately powered studies. Any observed interaction will require replication in larger, adequately powered samples.
